# Optic nerve assessments in MS diagnosis are worth the added implementation complexity: Yes

**DOI:** 10.1177/13524585251410417

**Published:** 2026-01-14

**Authors:** Gabriel Bsteh, Nik Krajnc

**Affiliations:** Department of Neurology, Medical University of Vienna, Vienna, Austria; Comprehensive Center for Clinical Neurosciences and Mental Health, Medical University of Vienna, Vienna, Austria; Department of Neurology, Medical University of Vienna, Vienna, Austria; Comprehensive Center for Clinical Neurosciences and Mental Health, Medical University of Vienna, Vienna, Austria

The 2024 revision of the McDonald criteria for multiple sclerosis (MS) represents a pivotal advancement in diagnostic precision by including the optic nerve as a fifth topographical region for dissemination in space (DIS).^1^ This revision acknowledges the visual pathway among the most frequently and earliest affected structures in MS. Optic nerve involvement can now be objectively demonstrated by orbital magnetic resonance imaging (MRI), optical coherence tomography (OCT), or visual evoked potentials (VEPs), each contributing complementary perspectives on demyelination and neuroaxonal loss.^2,3^ According to 2024 criteria, optic nerve involvement on orbital MRI requires a lesion visible within the optic nerve on coronal fat-suppressed T2-weighted or short tau inversion recovery (STIR) sequences, and/or on post-gadolinium-based contrast agent coronal fat-suppressed T1-weighted images.^2^ OCT-defined inter-eye differences (IED) of ⩾6 µm in peripapillary retinal nerve fibre layer (pRNFL) thickness or ⩾4 µm in macular ganglion cell-inner plexiform layer (GCIPL) thickness, and delayed or asymmetric VEP latencies relative to laboratory-specific norms, are also accepted indicators of optic nerve involvement when other causes are excluded.^3^ Integration of these modalities enhances diagnostic accuracy, accelerates diagnosis, and promotes equitable access.

In the McDonald 2024 framework, determining the number of DIS regions involved is fundamental.^1,4^ Recognizing optic nerve involvement can therefore be decisive for nearly all patients. For those presenting with optic neuritis (ON), comprising up to a third of patients, confirmation of optic nerve involvement provides one defined DIS region, necessitating only one additional region – in a combination of positive cerebrospinal fluid (CSF), positive central vein sign (CVS) or dissemination in time (DIT) – to reach diagnostic certainty.^1,4^ For those with subclinical optic nerve injury, observed in up to 50% of patients at diagnosis, optic nerve assessment may contribute a second region, completing the criteria for DIS, or even a fourth region, thereby enabling a diagnosis without the need for further paraclinical investigations.^5^ Robust evidence demonstrates that incorporating optic nerve metrics improves sensitivity without compromising specificity, increasing diagnostic accuracy from 66% to 81%.^6,7^ Thus, arguments that optic nerve assessment fails to provide meaningful clinical utility are rendered untenable.

Equally, concerns that these additions introduce undue diagnostic complexity are largely unfounded. Orbital MRI is the gold standard for confirming both symptomatic and asymptomatic MS-related optic nerve involvement, aiding differentiation from ischemic, compressive, or even inflammatory mimics.^2^ Conversely, conventional brain MRI is inadequate to reliably capture optic nerve involvement. Indeed, implementation of orbital MRI requires adaptation of existing MRI protocols, extends acquisition time and is not yet available to a considerable proportion of patients. However, OCT and VEP have long been integral to (neuro)ophthalmological and neurological routine practice, offering rapid, non-invasive, reproducible and cost-efficient assessments that can be seamlessly incorporated into existing diagnostic workflows. Together, these modalities offer a flexible and economically feasible diagnostic toolkit broadening the global applicability of the 2024 McDonald criteria, particularly in resource-limited settings.

The real strength of the new framework lies in combining complementary modalities to improve diagnostic specificity. In acute ON (<3 months), VEPs and orbital MRI provide the most sensitive combination, as VEP latency delay reflects active demyelination while orbital MRI confirms anatomical involvement. In remote ON (>3 months), orbital MRI and OCT are both highly sensitive in demonstrating structural optic nerve injury. In patients with no clear ON or in settings with limited MRI availability, the combination of OCT and VEP provides robust evidence of subclinical optic nerve involvement. These multimodal strategies are not redundant but mutually reinforcing. Concordant findings – OCT thinning with IED, VEP latency delay and a demyelinating MRI lesion – constitute compelling, objective evidence of optic nerve involvement.

As with all paraclinical tools, careful attention to quality control and interpretation is critical. Orbital MRI should employ standardized coronal fat-suppressed sequences and be assessed by experienced (neuro)radiologists. OCT acquisition should adhere to the OSCAR-IB criteria to ensure valid segmentation and artefact-free analysis, while VEPs should be performed using International Society for Clinical Electrophysiology of Vision standards, which notably include that each centre develops laboratory-specific normative datasets.^3^ Clinical context is indispensable: comorbidities such as uncontrolled diabetes or hypertension can influence retinal layer thickness and must be considered when interpreting OCT findings. Neither MRI nor OCT or VEP results are disease-specific; therefore, the principle of *no better explanation* remains central to assigning optic nerve lesions to MS. Comprehensive (neuro)ophthalmologic assessment, in conjunction with serological testing for aquaporin-4 (AQP4) and myelin oligodendrocyte glycoprotein (MOG) antibodies, provides the best way to rule out alternative causes.

Importantly, optic nerve assessment – particularly OCT – adds value beyond diagnosis. OCT-derived measures of pRNFL and GCIPL thinning correlate with relapse remission, brain atrophy, and long-term disability, offering a non-invasive biomarker for prognosis and monitoring.^8,9^ Longitudinal OCT can track neuroaxonal loss and treatment response over time.^10^ Hence, implementing assessment of (subclinical) optic nerve involvement not only facilitates diagnosis, but also strengthens risk stratification to aid therapeutic decisions.

Naturally, certain limitations remain. OCT IED thresholds are validated for unilateral ON but require further studies for bilateral involvement. VEP latency recovery following remyelination may obscure older lesions. Orbital MRI requires expertise and extends acquisition time, limiting its availability. Yet, these caveats are broadly analogous to those of other diagnostic tools and do not undermine the overall strength of the supporting evidence. With standardized acquisition protocols, rigorous quality control and interdisciplinary collaboration, optic nerve assessments can be safely, reliably and effectively integrated into routine clinical practice across healthcare systems.

In conclusion, the inclusion of the optic nerve as a fifth topographical region in the 2024 McDonald criteria is not an incremental refinement but a clinically pragmatic enhancement. Orbital MRI, OCT and VEP collectively provide anatomically and functionally complementary evidence of optic nerve injury, one of the most characteristic lesions in MS. Their judicious use increases diagnostic sensitivity, accelerates recognition of disease and extends diagnostic capacity to regions where advanced MRI is less accessible. The argument that adding optic nerve assessments creates unnecessary complexity does not withstand scrutiny: these modalities leverage technologies already embedded within neurology, ophthalmology and radiology practice. Implementation requires minimal workflow modification and can even foster multidisciplinary collaboration. The cumulative benefits clearly outweigh the nominal logistical demands. Rather than introducing complexity, optic nerve assessment brings MS diagnosis closer to biological truth, capturing the central role of visual pathway demyelination in the disease process. In contemporary, evidence-driven MS care, these assessments are not optional – they are essential.
